# Intensive versus standard physical rehabilitation therapy in the critically ill (EPICC): a multicentre, parallel-group, randomised controlled trial

**DOI:** 10.1136/thoraxjnl-2016-209858

**Published:** 2017-08-05

**Authors:** Stephen E Wright, Kirsty Thomas, Gillian Watson, Catherine Baker, Andrew Bryant, Thomas J Chadwick, Jing Shen, Ruth Wood, Jennifer Wilkinson, Leigh Mansfield, Victoria Stafford, Clare Wade, Julie Furneval, Andrea Henderson, Keith Hugill, Philip Howard, Alistair Roy, Stephen Bonner, Simon Baudouin

**Affiliations:** 1 Perioperative and Critical Care Directorate, Newcastle Upon Tyne Hospitals NHS Foundation Trust, Newcastle upon Tyne, UK; 2 Department of Physiotherapy, Newcastle Upon Tyne Hospitals NHS Foundation Trust, Newcastle upon Tyne, UK; 3 Newcastle Clinical Trials Unit, Faculty of Medical Sciences, Newcastle University, Newcastle Upon Tyne, UK; 4 Institute of Health & Society, Newcastle University, Newcastle Upon Tyne, UK; 5 Department of Anaesthetics, Sunderland Royal Hospital, City Hospitals Sunderland NHS Foundation Trust, Sunderland, UK; 6 Department of Anaesthetics, James Cook University Hospital, South Tees Hospitals NHS Foundation Trust, Middlesbrough, UK

**Keywords:** critical care, rehabilitation, early mobilisation, physiotherapy, mechanical ventilation

## Abstract

**Background:**

Early physical rehabilitation in the intensive care unit (ICU) has been shown to improve short-term clinical outcomes but long-term benefit has not been proven and the optimum intensity of rehabilitation is not known.

**Methods:**

We conducted a randomised, parallel-group, allocation-concealed, assessor-blinded, controlled trial in patients who had received at least 48 hours of invasive or non-invasive ventilation. Participants were randomised in a 1:1 ratio, stratified by admitting ICU, admission type and level of independence. The intervention group had a target of 90 min physical rehabilitation per day, the control group a target of 30 min per day (both Monday to Friday). The primary outcome was the Physical Component Summary (PCS) measure of SF-36 at 6 months.

**Results:**

We recruited 308 participants over 34 months: 150 assigned to the intervention and 158 to the control group. The intervention group received a median (IQR) of 161 (67–273) min of physical rehabilitation on ICU compared with 86 (31–139) min in the control group. At 6 months, 62 participants in the intervention group and 54 participants in the control group contributed primary outcome data. In the intervention group, 43 had died, 11 had withdrawn and 34 were lost to follow-up, while in the control group, 56 had died, 5 had withdrawn and 43 were lost to follow-up. There was no difference in the primary outcome at 6 months, mean (SD) PCS 37 (12.2) in the intervention group and 37 (11.3) in the control group.

**Conclusions:**

In this study, ICU-based physical rehabilitation did not appear to improve physical outcomes at 6 months compared with standard physical rehabilitation.

**Trial registration number:**

ISRCTN 20436833.

**Key message**

**What is the key question?**

Does an increased intensity of intensive care unit (ICU)-based physical rehabilitation therapy improve long-term physical quality of life compared with a standard intensity of physical rehabilitation as measured by the Physical Component Summary (PCS) measure of SF-36?

**What is the bottom line?**

Delivering more ICU-based physical rehabilitation did not appear to improve physical outcomes at 6 months but was limited by patient tolerance and a 5-day rehabilitation service.

**Why read on?**

The results of this trial of ICU-based physical rehabilitation therapy raise important questions about how early rehabilitation should be optimally delivered in the critically ill.

## Introduction

It has been recognised for some time that physical and psychological recovery after a period of critical illness is slow and often incomplete. Current evidence informs us that patients report ongoing physical and psychological problems, and a decreased quality of life, for up to 5 years after their original illness.^1^ These problems start early, with muscle wasting occurring during the first week of critical illness and being more severe among those with multiple organ failure.^2^ The aetiology of critical illness neuromyopathy is multifactorial, with both direct (toxic) and indirect (immobility/disuse atrophy) causes being implicated.^3^ Limiting the period of immobility and promoting movement and exercise are therefore intuitively attractive strategies to prevent muscle weakness and enhance recovery.

The concept of early mobilisation has developed in parallel with a better appreciation of the need to avoid over sedation, the importance of delirium and the value of spontaneous breathing trials.^4 5^ However, there are few randomised controlled trials of interventions delivered in the intensive care unit (ICU) on which to base clinical practice. In 2009, a landmark trial by Schweickert and colleagues found that early physical and occupational therapy in mechanically ventilated patients in the medical ICU was safe and well tolerated, and resulted in better functional outcomes at hospital discharge, a shorter duration of delirium and more ventilator-free days compared with standard care.^6^ A recent multicentre trial in the surgical ICU also found that early, goal-directed mobilisation shortened patient length of stay and improved patients’ functional mobility at hospital discharge.^7^

However, longer-term benefits have not yet been established^8–10^ and systematic reviews have identified a need for large randomised controlled trials to further explore early mobilisation therapy, including longer-term outcomes and the ideal intensity and timing of exercise.^11 12^ Our hypothesis was that patients would report a sustained benefit in their physical health if they received more intensive physical rehabilitation in ICU compared with patients who received physical rehabilitation typical of that provided at the time. In an attempt to test this hypothesis, we undertook a randomised controlled trial comparing the effects of two different intensities of early rehabilitation therapy—intensive versus standard—on the recovery of physical health-related quality of life at 6 months.

## Methods

### Study design

We conducted a randomised, parallel group, allocation-concealed, assessor-blinded, controlled trial in mixed medical-surgical ICUs of four hospitals in the UK. The trial had ethical approval from Newcastle and North Tyneside 2 Research Ethics Committee (11/NE/0206) and was registered (ISRCTN: 20436833). A full trial protocol has been published.^13^

### Participants

Patients were eligible for inclusion in the trial if they were aged 18 years or older and had received 48 hours or more of either invasive or non-invasive ventilation. The original (and registered) version of the protocol listed 48–72 hours but the upper limit was removed 2 months into recruitment as the time window proved too narrow and eligible patients were being missed, especially at weekends. The exclusion criteria were as follows: end-of-life care; acute brain or spinal cord injury (or admitted following brain or spinal cord surgery); multiple trauma if mobilisation therapy was judged unlikely to be possible; burns; rapidly progressive neuromuscular disease; patients enrolled in another clinical trial without a co-enrolment agreement in place; and patients previously enrolled in this trial. Patients who had suffered a cardiac arrest could be recruited if the clinical team believed that there was a possibility of recovery. Patients were approached and written informed consent was obtained either from the patient or from a personal consultee, which was usually the next of kin.

### Randomisation and blinding

Participants were randomised, using a web-based randomisation system, in a 1:1 ratio, using permuted random block allocation to either intervention or standard care group. Randomisation was stratified by admitting ICU, type of admission (surgical or medical) and the participant’s prehospitalisation independence level using the Katz Index of independence in activities of daily living (0–3 was ‘low’, 4–6 was ‘high’). Owing to the nature of the intervention, it was not possible to mask the participants, research physiotherapists or the wider clinical team to the treatment group. Study outcomes at ICU discharge were assessed by the research physiotherapists (unblinded). Study outcomes at hospital discharge and at 3 and 6-month follow-up were assessed by research nurses who were blinded to the treatment group. To avoid accidental unblinding, the research nurses would ask the participants not to reveal their treatment group.

### Procedures

All physical rehabilitation sessions were preceded by a sedation hold (or titration) with a target Richmond Agitation-Sedation Scale of −1, 0 or +1 and a safety screen.^14^ The physical rehabilitation therapy provided in the study included functional training and individually tailored exercise programmes.^13^ All physical rehabilitation therapy was provided by experienced critical care physiotherapists. The intervention group had a target delivery of 90 min of physical rehabilitation per day (Monday to Friday), split between at least two sessions. The standard care group had a target of 30 min of physical rehabilitation per day (Monday to Friday). Days when physical rehabilitation was able to be delivered were termed ‘treatment days’. The session was stopped immediately if the participant met any of the stopping criteria; otherwise, the session continued until either the target time was reached or the treating physiotherapist judged it appropriate to stop. The physical rehabilitation therapy received by the standard care group was the same as that provided normally in participating ICUs.^15^ Respiratory physiotherapy was given as standard in both groups.

The treating physiotherapists collected data on the time participants were actively engaging with physical rehabilitation and the type of exercise/mobilisation completed; these times do not include the time required for session set-up and preparation, rest and recovery between exercises or passive range of movements. They also recorded the maximum strength demonstrated in both upper and lower limbs and/or the maximum mobility level reached during each session. Limb strength was measured using the Oxford Scale. The mobility levels achieved were defined as follows: (1) unable to sit supported; (2) able to sit supported; (3) able to sit out in a chair safely; (4) able to stand to transfer with support; (5) able to mobilise independently.^13^

Following discharge from ICU, both groups received routine ward-based physiotherapy and an exercise diary to continue independently on discharge from hospital. Any participant readmitted to ICU received standard physical rehabilitation during their first and any subsequent readmissions. Participants were followed until hospital discharge and invited to attend a follow-up at their hospital 3 and 6 months after randomisation. Follow-up questionnaires could be completed by telephone if participants were unable to attend in person.

### Outcomes

The primary outcome was the Physical Component Summary (PCS) measure of the 36 item Short Form survey (SF-36) (version 2) Quality of Life questionnaire at 6 months.^16^ Secondary outcomes were as follows: the Mental Health Component Summary (MCS) measure of the SF-36; physical ability at ICU discharge (Modified Rivermead Mobility Index)^17^; length of ICU and hospital stay; exercise capacity (6 min walk test)^18^; functional status (Functional Independence Measure)^19^; hand grip strength; and survival status and place of residence at 3 and 6 months following randomisation. The health economic evaluation used utility values derived from the EuroQol 5 dimension survey (EQ-5D)^20^ (administered at hospital discharge, 3 and 6 months) and from the SF-36, using the algorithm provided by the SF-6D.^21^ Survival data up to 6 months were recorded for all participants from the Health & Social Care Information Centre.

### Statistical analysis

A sample size calculation based on a difference of five points in the primary outcome (PCS of SF-36), 80% power and a significance level of 0.05 required 77 patients to contribute primary outcome data at 6 months. Allowing for a mortality rate of 40% and a further 10% loss to follow-up required 154 in each group (308 participants in total). We used univariate analysis to calculate basic summary statistics and multiple linear regression to adjust for the effects of covariates, including stratification variables. Kaplan-Meier and the log-rank test were used to compare survival between the groups at ICU and hospital discharge as well as at 3 and 6 months. Cox proportional hazards models were used to compute unadjusted and adjusted HRs for overall survival. All analyses were performed on an intention-to-treat basis. A statistical analysis plan was written and agreed by the Trial Management Group before analysis of the study data. Statistical analysis was performed using STATA V. 14. Additional descriptive analysis of physiotherapy data (table 1) was carried out using Microsoft Excel by an external consultant overseen by the Trial Management Group.

**Table 1:** Characteristics of physical rehabilitation delivered at group and participant level

	Intensive (n=150)	Standard care (n=158)
Group-level data		
Physical rehabilitation sessions		
Functional retraining Strengthening Functional retraining and strengthening Total	757 (37%) 674 (33%) 637 (31%) 2068	488 (37%) 406 (30%) 441 (33%) 1335
Physical rehabilitation time, min		
Functional retraining Strengthening Total time	15 032 12 317 27 349	9110 6269 15 379
Participant-level data		
Days from enrolment to first day when physical rehabilitation received	3 (1–6)	3 (1–6)
Physical rehabilitation time in ICU (total received), min		
Functional retraining Strengthening Total time	86 (31–154) 72 (28–123) 161 (67–273)	45 (20–82) 24 (9–55) 86 (31–139)
Physical rehabilitation sessions in ICU	10 (4–19)	6 (2–12)
Percentage study days where physical rehabilitation received*	57 (34–77)	40 (17–54)
Physical rehabilitation time per treatment day†, min		
Functional retraining Strengthening Total time	12 (8–17) 10 (6–15) 23 (16–28)	8 (5–10) 5 (2–8) 13 (10–17)

Data are n (%) or median (IQR).

*Study days are defined as the number of days between enrolment and the date the participant was recorded as being ready for discharge from ICU; if unavailable, the actual date of ICU discharge was used.

†Treatment days are defined as the number of days when the participant received physical rehabilitation on ICU; physical rehabilitation in either arm was only delivered Monday to Friday. Data are ‘median of means’, that is, a mean time was calculated for each participant and then, as the data were not normally distributed, a median (IQR) was calculated for each trial arm.

ICU, intensive care unit.

The health economic analysis compared costs of both the standard care and intervention groups from the health service perspective as well as a societal perspective. Patient costs for hospital visits were assessed at 6 months using the Patient Costs Questionnaire.^13^ Utility scores were calculated from SF-6D derived from responses to SF-36 and EQ-5D collected at 3 and 6 months after hospital discharge. For the purpose of calculating quality-adjusted life years (QALYs), baseline utility values were assumed to be the same value for all participants on admission to ICU. For each participant, we used the lowest possible value for SF-6D, the value for EQ-5D was based on a combination of responses to the questionnaire (mobility-3, selfcare-3, usual activities-3, pain/discomfort-2, anxiety-2) that was assumed to be appropriate for the average patient admitted to ICU. QALYs were calculated using the area under the curve method with three time points of baseline and 3 and 6 months.^22^

## Results

**Figure 1:**
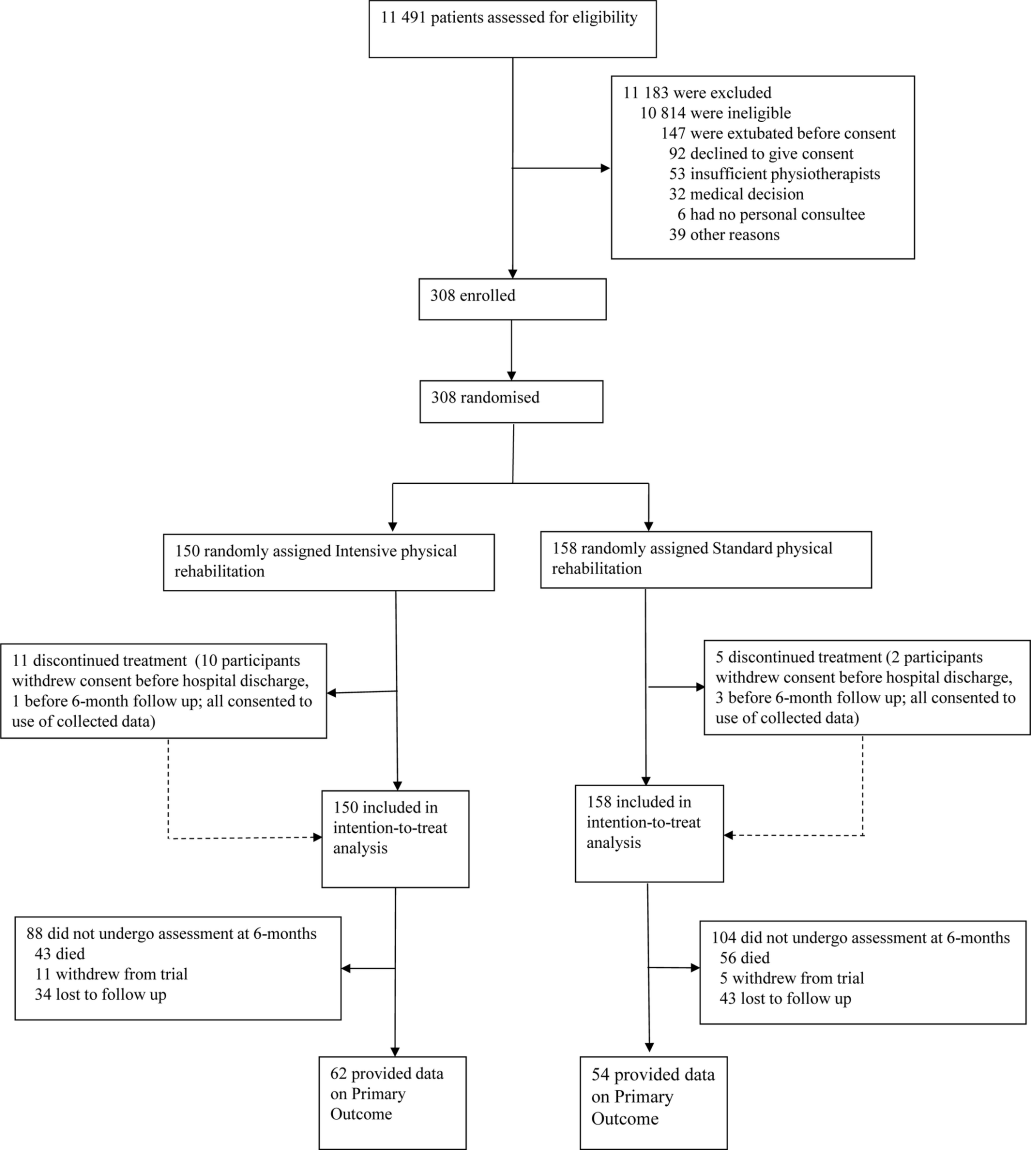
Trial profile.

Between 16 January 2012 and 4 December 2014, we enrolled 308 participants, 150 were assigned to receive intensive and 158 to receive standard physical rehabilitation therapy (figure 1). Sixteen participants withdrew consent, but all consented to the use of collected data. At 6 months, 62 (41%) participants in the intervention group and 54 (34%) participants in the standard care group contributed primary outcome data, a total of 116 of the 154 (75%) expected from the original sample size calculation. In the intervention group, 43 (29%) had died, 11 (7%) had withdrawn and 34 (23%) were lost to follow-up, while in the standard care group, 56 (35%) had died, 5 (3%) had withdrawn and 43 (27%) were lost to follow-up. Including participants who had withdrawn from the study in the denominator, primary outcome data were collected for 62 of 107 (58%) participants alive at 6 months in the intervention group, and 54 of 102 (53%) participants alive at 6 months in the standard care group.

The study groups had similar baseline characteristics (table 2) although the proportion of males in the intensive group (54%) was lower than in the standard care group (63%). The majority of participants in both groups were admitted to ICU as an emergency rather than as planned postoperative admissions. Participants in both groups were divided equally between medical and surgical admissions, and had similar severity of illness scores. Prehospitalisation independence levels were high, with 135 (93%) of the intensive group and 144 (93%) of the standard care group being functionally independent (Katz Index 6) at baseline. Before randomisation, participants in the intervention group had been on ICU for a median (IQR) of 6 (4–9) days compared with 5 (4–8) in the standard care group. Participants in both groups had been ventilated for a similar number of days before randomisation (median 4 days in both groups).

**Table 2:** Characteristics of study participants at baseline

	Intensive (n=150)	Standard care (n=158)
Age (years)	60 (16)	64 (16)
Sex		
Male Female	81 (54%) 69 (46%)	99 (63%) 59 (37%)
Type of admission		
Emergency Planned	134 (90%) 16 (10%)	143 (90%) 15 (10%)
Specialty		
Medical Surgical	78 (52%) 72 (48%)	82 (52%) 76 (48%)
APACHE II score	19 (7)	19 (7)
ICNARC Physiology Score	22 (8)	23 (9)
Premorbid Katz Index		
Low score (0–3) High score (4-6)	4 (3%) 141 (97%)	6 (4%) 149 (96%)
ICU length of stay (days)*	6 (4–9)	5 (4–8)
Duration of ventilation (days)*	4 (3–7)	4 (3–6)
Mode of ventilation		
Invasive Non-invasive	148 (99%) 1 (1%)	153 (98%) 4 (2%)

*ICU length of stay and duration of ventilation are at time of randomisation.

Data are n (%), mean (SD) or median (IQR).

APACHE, Acute Physiology and Chronic Health Evaluation; ICNARC, Intensive Care National Audit and Research Centre; ICU, intensive care unit.

In the intensive group, physiotherapists attempted to deliver 4079 physical rehabilitation sessions, of which a sedation hold (or titration) was judged safe and undertaken in 3579 (87.7%). Participants had a successful sedation hold (or titration), passed the safety screen and started physical rehabilitation in 2068 (50.7%) sessions in the intensive group. In the standard care group, physiotherapists attempted to deliver 2515 physical rehabilitation sessions, of which a sedation hold (or titration) was judged safe and undertaken in 2141 (85.1%). Participants had a successful sedation hold (or titration), passed the safety screen and started physical rehabilitation in 1335 (53.1%) sessions in the standard care group.

**Figure 2:**
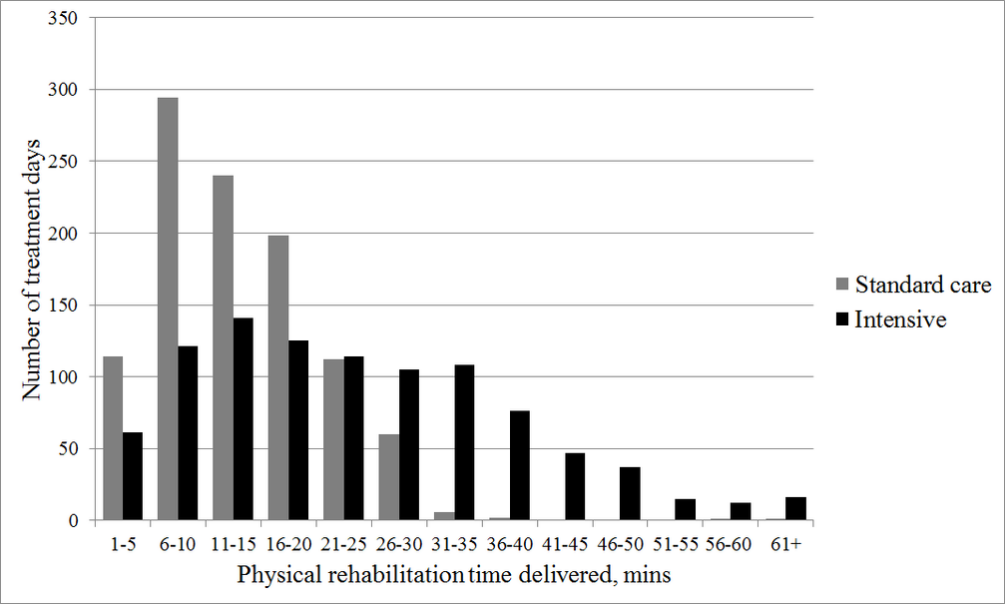
Physical rehabilitation time delivered per treatment day. Treatment days are defined as the number of days when the participant received physical rehabilitation on intensive care unit.

Details of the physical rehabilitation sessions delivered in the two study groups are shown in table 1. At a participant level, the median (IQR) number of days from enrolment to the first day when physical rehabilitation was received (excluding passive range of movement exercises) was 3 (1–6) days in both groups. In total, the intervention group received a median (IQR) of 161 (67–273) min of physical rehabilitation on ICU compared with 86 (31–139) min in the standard care group. The majority of the extra time was given to strengthening exercises over functional retraining. Physical rehabilitation was delivered on 57% of study days in the intervention group and 40% of study days in the standard care group. Study days were defined as the number of calendar days between the date of enrolment and the date a participant was recorded as being ready for ICU discharge (if unavailable, the actual date of ICU discharge was used) and therefore include weekends, when no physical rehabilitation service was provided. On days when physical rehabilitation was able to be delivered (‘treatment days’), the intervention group received a median (IQR) of 23 (16–28) min of physical rehabilitation per day compared with 13 (10–17) min per day in the standard care group. The distribution of physical rehabilitation time delivered on treatment days differed between two groups (figure 2). The majority of treatment days in the standard care group included less than the target of 30 min physical rehabilitation, with 82% of treatment days including 20 min or less. In the intervention group, which had a target delivery of up to 90 min per day, the time delivered was more variable but only 8% of treatment days included greater than 45 min of physical rehabilitation.

**Figure 3:**
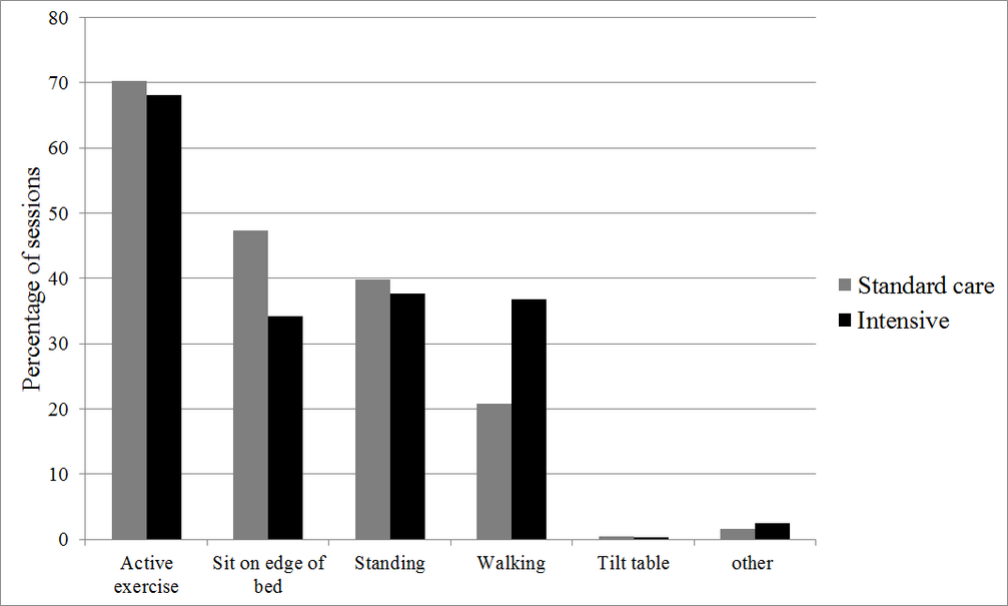
Content of physical rehabilitation sessions.

**Figure 4:**
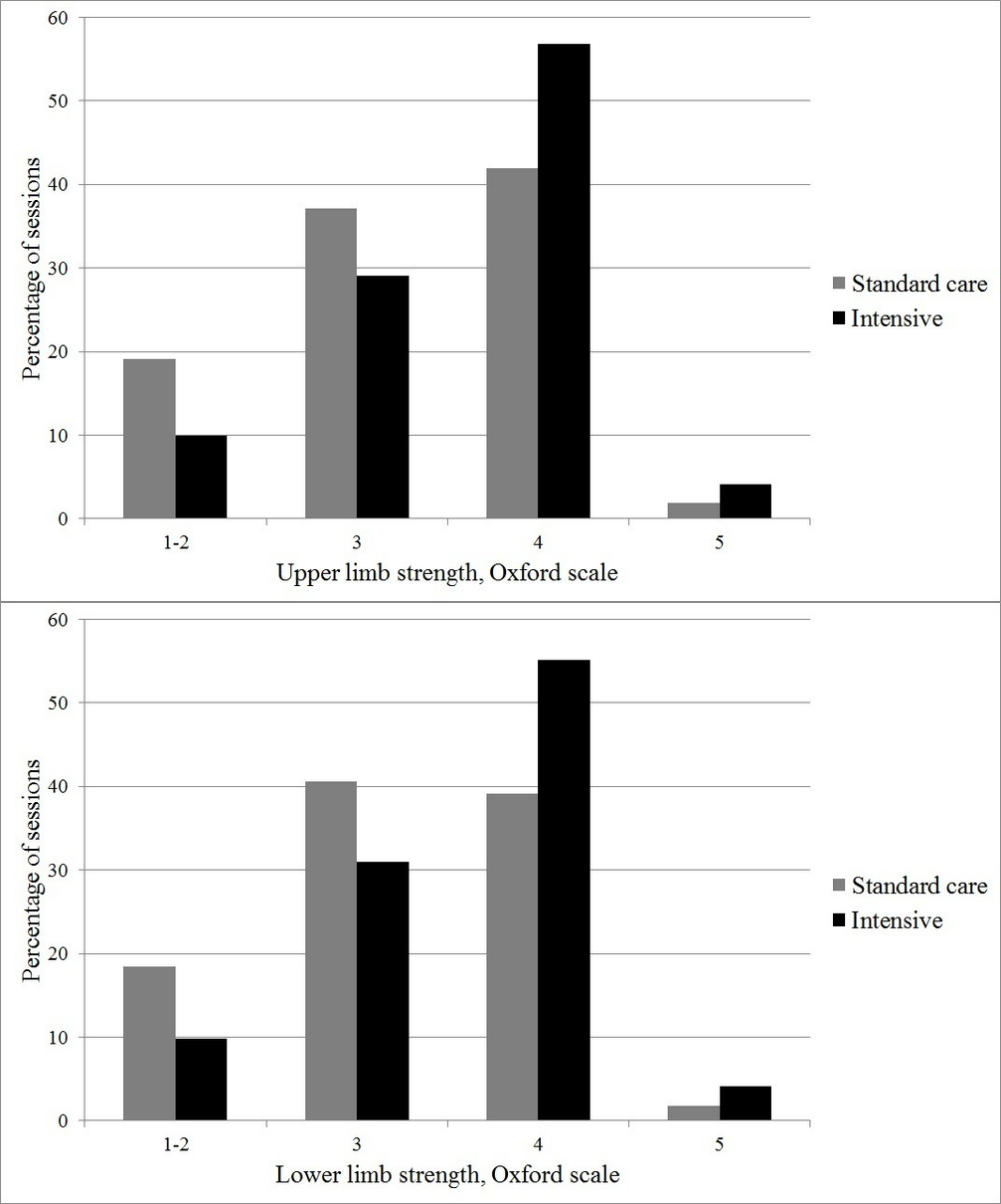
Upper and lower limb strength achieved during physical rehabilitation sessions. Oxford Scale: 0/5 no contraction; 1/5 visible/palpable muscle contraction but no movement; 2/5 movement with gravity eliminated; 3/5 movement against gravity only; 4/5 movement against gravity with some resistance; 5/5 movement against gravity with full resistance.

**Figure 5:**
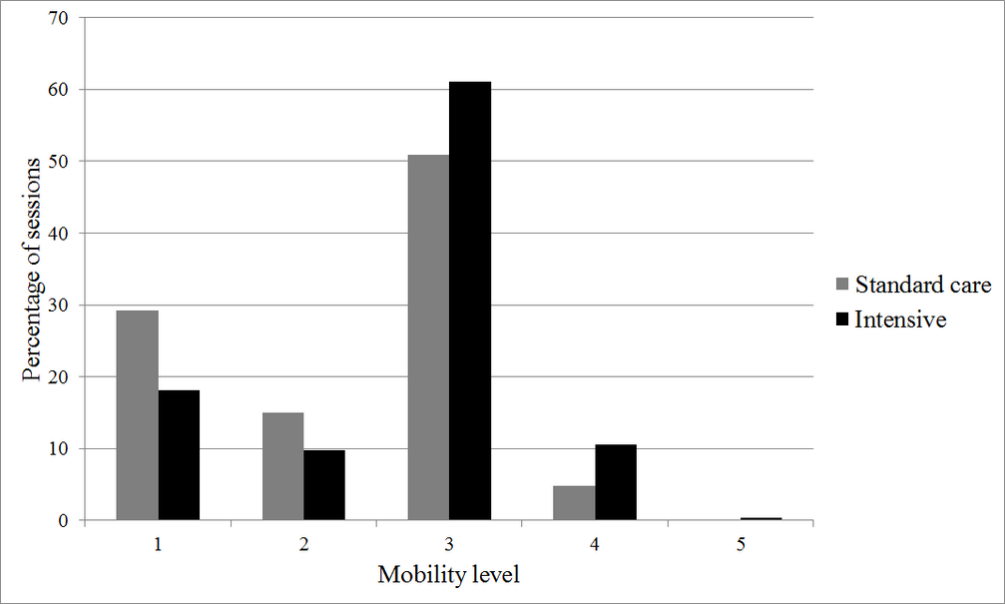
Mobility level achieved during physical rehabilitation sessions.

The content of the individual physiotherapy sessions was broadly similar between groups, although a greater percentage of sessions included walking in the intervention arm (figure 3). With regard to limb strength, a greater proportion of sessions included exercises at Oxford Scale 4 or 5 in the intervention arm than in the standard care arm (figure 4). With regard to mobility, a greater proportion of sessions included mobilisation to levels 3, 4 or 5 in the intervention arm than in the standard care group (figure 5).

**Figure 6:**
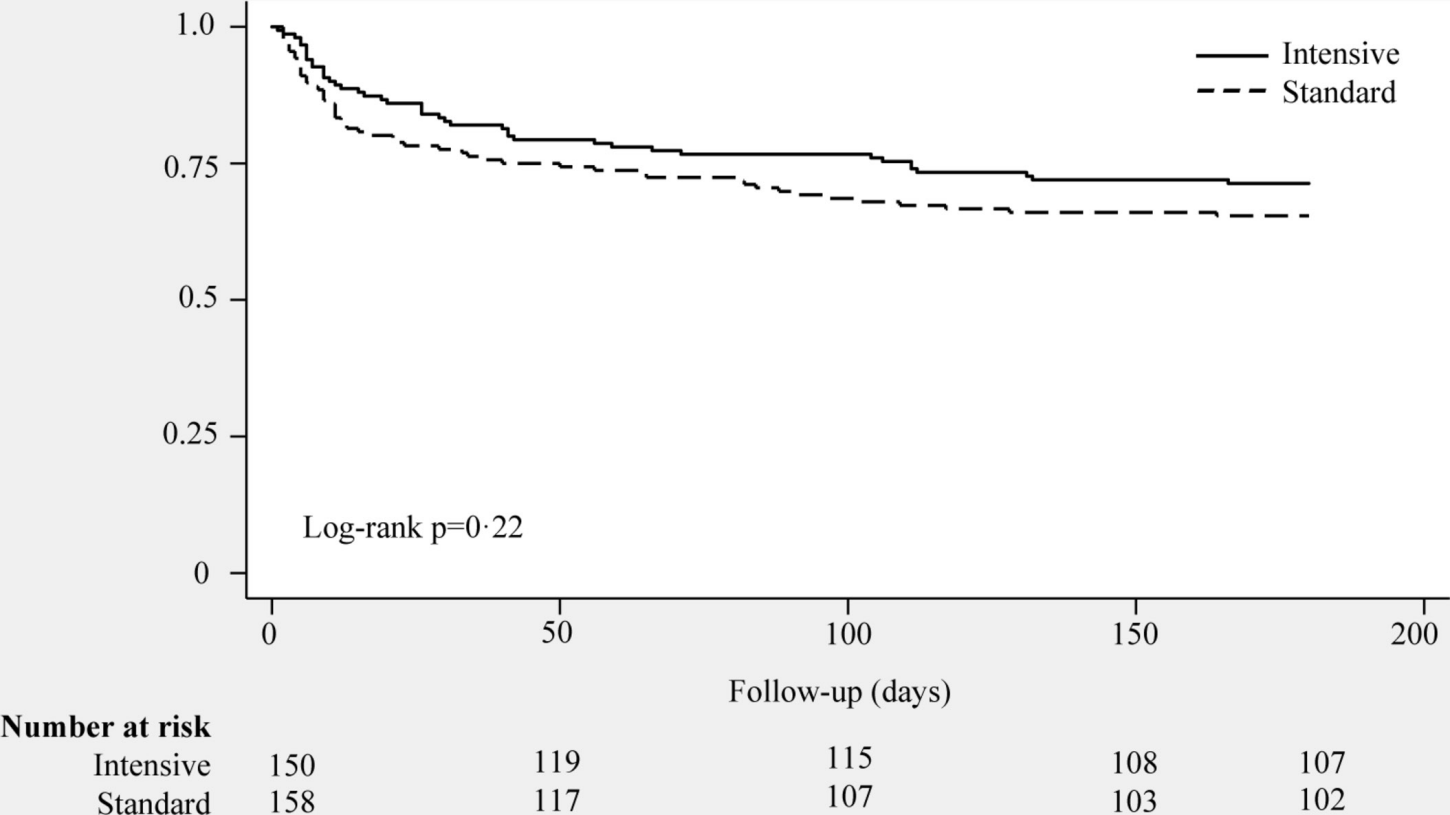
Participant survival to 6 months postrandomisation.

The primary outcome measure, the mean (SD) PCS measure of the SF-36 at 6 months, was 37 (12.2) in the intervention group and 37 (11.3) in the standard care group with an adjusted difference in means −1.1 (95% CI −7.1 to 5.0). Secondary outcomes were also similar between groups across all follow-up time points (table 3 and online supplementary table S1). Only one secondary outcome, the Functional Independence Measure at 3 months, was significantly different between groups, although this should be interpreted with caution given the multiple tests. With regard to the loss to follow-up, there did not appear to be any differences in baseline characteristics between participants who were and those were not able to complete the primary outcome measure (online supplementary table S2). We undertook a post hoc analysis using multiple imputation for the primary outcome measure but the results were the same (online supplementary table S3). There was no significant difference in overall survival between groups at any time in the 6-month follow-up period (figure 6). This analysis was robust to adjusted and unadjusted Cox proportional hazards regression. By 6 months, most participants had been discharged home, although seven in each group remained in hospital.

10.1136/thoraxjnl-2016-209858.supp1 Supplementary file 1

**Table 3:** Primary and secondary outcome measures

	Intensive n=150		Standard care n=158		Difference*
	Physical Component Summary measure of SF-36				
Hospital discharge	n=76/116	34 (8.0)	n=76/113	34 (9.0)	−1.0 (−4.2 to 2.2)
3 months	n=69/115	35 (10.8)	n=69/109	34 (9.0)	−1.4 (−5.9 to 3.0)
6 months	n=62/107	37 (12.2)	n=54/102	37 (11.3)	−1.1 (−7.1 to 5.0)
	Mental Component Summary measure of SF-36				
Hospital discharge	n=76/113	40 (13.2)	n=76/113	39 (11.4)	1.3 (−3.4 to 6.0)
3 months	n=69/115	47 (11.8)	n=69/109	45 (12.3)	4.2 (−1.2 to 9.5)
6 months	n=62/107	47 (15.0)	n=54/102	48 (11.5)	−0.4 (−6.5 to 5.7)
	Modified Rivermead Mobility Index†				
ICU discharge	n=112/124	19 (10)	n=104/118	16 (10)	0.4 (−2.7 to 3.4)
	ICU length of stay (median (IQR) days)				
Participants alive at ICU discharge	n=124	13 (8–21)	n=118	15 (8–23)	0.3 (−4.0 to 4.8)‡
Participants deceased at ICU discharge	n=26	10 (6–29)	n=40	9 (4–14)	5.2 (−7.3 to 16.7)‡
	Hospital length of stay (median (IQR) days)				
Participants alive at hospital discharge	n=116	28 (19–56)	n=113	28 (20–43)	4.1 (−6.5 to 15.3)‡
Participants deceased at hospital discharge	n=34	14 (6–31)	n=45	9 (5–21)	−5.0 (−29.2 to 14.7)‡
	Six-minute walk test (median (IQR) in metres)				
Hospital discharge	n=49/116	195 (120–260)	n=34/113	173 (123–274)	−27.9 (−86.1 to 31.8)‡
3 months	n=32/115	293 (124–444)	n=27/109	255 (120–337)	−6.3 (−125.8 to 107.3)‡
6 months	n=28/107	374 (203–435)	n=25/102	321 (197–400)	61.7 (−47.2 to 157.0)‡
	Functional Independence Measure§				
ICU discharge	n=114/124	70 (27)	n=107/118	64 (25)	−0.6 (−7.1 to 8.3)
Hospital discharge	n=83/116	113 (17)	n=75/113	108 (20)	1.2 (−5.2 to 7.7)
3 months	n=71/115	116 (19)	n=67/109	111 (23)	9.7 (0.9 to 18.5)
6 months	n=64/107	118 (17)	n=51/102	117 (16)	3.7 (−5.4 to 12.7)
	Grip strength				
ICU discharge	n=95/124	14 (9)	n=89/118	15 (9)	−0.6 (−3.3 to 2.1)
Hospital discharge	n=60/116	19 (10)	n=54/113	22 (14)	−3.5 (−8.1 to 1.2)
3 months	n=36/115	25 (16)	n=31/109	24 (14)	1.8 (−6.7 to 10.3)
6 months	n=31/107	29 (19)	n=31/102	24 (16)	−0.5 (−11.3 to 10.3)

*Adjusted difference in means (95% CI). Multiple linear regression models included stratification variables (unit, admission type and preadmission Katz Index) and baseline variables sex, mode of ventilation, specialty, age in years, body mass index, randomisation lag (time between admission to ICU and participant randomisation), duration of ventilation and ICNARC Physiology Score.

†Distribution was positively skewed so bootstrap sampling was used to estimate 95% CIs for adjusted difference in means.

‡The Functional Independence Measure rates patients on a scale of 1 (<25% independence; total assistance required) to 7 (100% independence) against 18 activities, giving a maximum score of 126.

§The Modified Rivermead Mobility Index rates patients on a scale of 0 (unable to perform) to 5 (independent) against eight physical tasks, giving a maximum score of 40.

ICNARC, Intensive Care National Audit and Research Centre; ICU, intensive care unit.

Results of the health economic analysis are shown in online supplementary table S4. Resource use during participants’ primary hospital admission was greater in the intervention group due to the increased physiotherapist time. Utility scores and QALYs were similar between groups.

One adverse event (AE) related to physical rehabilitation was reported during the study and occurred in the intervention group when a tracheostomy needed to be re-sited for a cuff leak. A second AE was reported in this group; however, on review this was found to have occurred well outside the relevant reporting time period. There were no serious AEs.

## Discussion

In this randomised controlled trial, a regime which provided more intensive physical rehabilitation in the critically ill did not confer any additional benefit over standard physical rehabilitation. We found no difference in either the primary outcome of self-reported physical health at 6 months or the secondary outcomes, including measures of functional ability and independence, length of ICU and hospital stay, and mortality at 6 months. Our results are similar to those of the two other randomised trials that included long-term assessments of physical function.^9 23^

This study has three main limitations. First, the absolute difference in the amount of physical rehabilitation received by participants in each arm of the trial was smaller than anticipated and possibly insufficient to produce a measurable difference in outcome.^24^ This was due to both the amount of physical rehabilitation received in the standard care group and factors that prevented more rehabilitation being delivered in the intensive group—the two principal reasons being a difficulty in achieving optimum levels of sedation (to allow rehabilitation to proceed safely) and participant fatigue. Most rehabilitation sessions ended due to participant fatigue, either at patient request or at the decision of the physiotherapist. Staff availability was not a major factor in understanding why the intervention was not delivered as intended as physiotherapists were available Monday to Friday. Second, we underestimated the difficulty in following up survivors of critical illness beyond discharge from hospital; only 116 of a planned 154 participants (75%) were able to contribute primary outcome data at 6 months. This proportion of loss to follow-up reduces the precision of the results and could introduce bias, although the baseline characteristics of those who did and did not complete the SF-36 questionnaire at 6 months were similar. There is also a variable amount of missing data for the secondary outcomes as those completing follow-up by telephone were unable to complete the 6 min walk test and grip strength assessment. Third, it was not possible to blind participants, physiotherapists or other clinical staff to the intervention, which could have introduced bias.

This study also has several strengths. Compared with the existing literature it is relatively large; to our knowledge only the recent, single-centre trial by Morris and colleagues is of a similar size.^25^ Although the absolute difference between groups was smaller than anticipated, the intervention group did receive a more intensive physical rehabilitation regime than the standard care group. Despite the loss to follow-up, the majority of participants underwent comprehensive assessments at ICU discharge, and we collected complete data for ICU and hospital length of stay and mortality to 6 months. Finally, the research staff undertaking assessments at, and after, hospital discharge were blinded to the study group.

It is useful to compare our findings with those of the other published trials. An important study by Denehy *et al* 9 was unable to show an improvement in outcome at 6 months, probably because the usual care group received a high level of physical mobilisation therapy.^24^ Participants in our standard care group received physical rehabilitation on 40% of study days, with 70% of rehabilitation sessions including active exercises, a greater intensity of physical rehabilitation than the ‘usual care’ recorded in other countries.^26 27^ On the other hand, participants in the intervention group received physical rehabilitation on 57% of study days an identical percentage to that reported in the intervention group by Morris *et al* 25 but less than that reported by others.^23 28^ Although physical rehabilitation times are not widely reported in other trials, the intervention group received a median (IQR) of 23 (16–28) min which is broadly comparable to the mean (SD) ‘session times’ of 31 (7) min in the study by Moss *et al* 23 and the median (IQR) 20 (0–41) min of ‘active exercises’ received in a pilot study by Hodgson *et al*.^28^ The proportion of patients lost to follow-up at 6 months is similar to that reported by Moss *et al* 23 at the same time point and also by Schaller *et al* at 3 months.^7^ Other trials, however, have achieved much better rates of follow-up to 6 months.^9 28 29^ Finally, a large proportion of screened patients were ineligible for the trial, most commonly because they had not received 48 hours or more of either invasive or non-invasive ventilation, although other exclusion criteria applied. Because of the size of our trial, we chose eligibility criteria aiming to include patients at high risk of physical impairment following discharge from ICU while excluding those who would be unlikely to benefit from the intervention. The result was a trial population which represented a relatively small subset of critically ill patients. The proportion of exclusions in other rehabilitation trials varies but the trial by Morris *et al* 25 also reported a high proportion of exclusions with 4804 patients screened for 300 patients randomised.

There are five other possible explanations for why our results did not support our hypothesis.

1. The patient population—which was heterogeneous, older, with a longer duration of mechanical ventilation—may have been predicted to have the worst levels of disability after critical illness and, on the whole, have been unresponsive to the intervention.^30^ The primary outcome measure, which showed wide variation within groups, was flat for the first 3 months and increased slowly in both groups by 6 months. A very similar recovery trajectory was seen in a cohort study of patients recovering from severe acute pancreatitis^31^ and in the RECOVER trial.^29^ In contrast, the recovery trajectory in a large cohort study of younger survivors with acute respiratory distress syndrome (ARDS) was more rapid, with an early improvement in the same PCS outcome measure.^1^
2. It is possible that the intervention was not started early enough and that muscle weakness was already well established.^2^ The median (IQR) duration of ventilation at randomisation was 4 (3–7) days with a further 3 (1–6) days until the first physical rehabilitation was received, excluding passive range of movements. In our experience, these delays occurred for a number of reasons: we did not have the resources to recruit patients at weekends; the eligibility criteria required patients to received 48 hours or more of either invasive or non-invasive ventilation before randomisation, although we usually approached surrogate decision makers before this; and in patients who were recruited, many were too unstable to achieve the required level of sedation and pass the safety screen to begin physical rehabilitation. This timescale from ICU admission, to enrolment, to active rehabilitation is comparable to some studies,^8 9 23 25^ although other investigators have succeeded in delivering much earlier rehabilitation therapy.^6 7 28^ The results from previous studies are not consistent but it would appear that the trials which have managed to deliver very early mobilisation have found improved outcomes up to hospital discharge,^8 9^ while trials which intervened later have found no significant effect. The exceptions to this observation are the trial by Burtin *et al* 8 which recruited patients from their fifth day in ICU and found improved physical outcomes at hospital discharge and the trial by Morris *et al* 25 which recruited patients in a similar timescale to our trial and found improved physical outcomes at 6 months.
3. It is also possible that the intervention ended too soon and that any benefits gained on ICU were subsequently lost on the ward or following discharge from hospital; however, this is not supported by the results which show no difference in physical or functional scores at ICU discharge.
4. Our statistical analysis did not specifically deal with the competing risk of mortality, which was non-significantly higher in the standard care group at 3 and 6 months. Although unlikely to be an important source of bias, it is possible that an intervention which improves survival could lead to a number of survivors with a low health-related quality of life.
5. The PCS measure of the SF-36 may not have been sensitive to change as it includes scoring coefficients from all eight domains of the SF-36, including the negative-scoring coefficients ‘role-emotional’ and ‘emotional well-being’, which may not have been affected by the intervention.^32^

Further improvements in physical rehabilitation will require a better understanding of the different phenotypes of critical illness and their varying recovery trajectories. Rehabilitation may then be better tailored to the individual, with perhaps the most intensive therapy aimed at younger patients, with less comorbidity, who have seen the biggest loss in function compared with baseline. New technology may enable patients to tolerate more physical rehabilitation in a way that fits flexibly with their other needs. Future work is needed to explore the barriers and facilitators to patients attending follow-up appointments, as well as ongoing work to determine the best outcome measures for rehabilitation trials.

In conclusion, this study set out to test whether an increased intensity of physical rehabilitation above that already delivered as standard in our region could lead to improved long-term patient outcomes. We were not assessing some physical rehabilitation versus no physical rehabilitation but rather ‘intensive’ versus ‘usual care’, both of which included care delivered by specialist critical care physiotherapists 5 days a week. It proved more difficult than expected to increase the intensity of rehabilitation above this baseline, due to participant fatigue and the inability to provide rehabilitation at weekends; this may have limited the impact of the intervention. The loss to follow-up at 6 months may have introduced bias and limits the confidence in our findings. In this context, ICU-based physical rehabilitation did not appear to improve physical outcomes at 6 months compared with standard physical rehabilitation.
