# Supplementary material for: Intensive versus standard physical rehabilitation therapy in the critically ill (EPICC): a multicentre, parallel-group, randomised controlled trial
Source: Thorax. 2017 Aug 5;73(3):213–21. doi: 10.1136/thoraxjnl-2016-209858 (PMC5870467; doi:10.1136/thoraxjnl-2016-209858)
Supplement: Supplementary file 1 [file thoraxjnl-2016-209858supp001.pdf]

Supplementary material

Intensive versus standard physical rehabilitation therapy in the critically ill (EPICC): a multi-centre, parallel group, randomised controlled trial

Page

Page	Item	Description
2.	S1	Secondary outcomes: body-mass index.
3.	S2	Characteristics of study participants at baseline in participants who were and were not able to complete the Physical Component Summary measure of SF-36 at six months.
4.	S3	Primary outcome measure after multiple imputation.
5.	S4	Health economic analysis.
6.		A survey to evaluate the provision of critical care physiotherapy services in the North East of England. Abstract presented at Chartered Society of Physiotherapists Congress, Liverpool, 15-16 October 2010.

Table S1 Secondary outcomes: body-mass index.

	Intensive		Standard care		Difference*
	Body-mass index (kg/m^2)				
ICU discharge	n=52/124	26 (7)	n=47/118	28 (7)	-0.6 (-2·5–1.3)
Hospital discharge	n=64/116	26 (7)	n=57/113	26 (7)	0.8 (-0·8–2·5)
3 months	n=41/115	27 (7)	n=37/109	27 (9)	-0.6 (-4·0–2·9)
6 months	n=39/107	27 (7)	n=35/102	28 (8)	-0.8 (-4·4–2·9)

* Adjusted difference in means (95% CI). Multiple linear regression models included stratification variables (unit, admission type and pre-admission Katz Index) and baseline variables sex, mode of ventilation, specialty, age in years, randomisation lag (time between admission to ICU and participant randomisation), duration of ventilation, ICNARC Physiology Score and body-mass index (kg/m^2).

Table S2 Characteristics of study participants at baseline in participants who were and were not able to complete the Physical Component Summary measure of SF-36 at six months.

	Able to complete (n=115)	Unable to complete, excluding deceased (n=94)
Age (years)	63 (14)	56 (18)
Sex		
Male	62 (54%)	57 (61%)
Female	53 (46%)	37 (39%)
Type of admission		
Emergency	103 (90%)	84 (89%)
Planned	12 (10%)	10 (11%)
Speciality		
Medical	63 (55%)	47 (50%)
Surgical	52 (45%)	47 (50%)
APACHE II score	18 (7)	18 (7)
ICNARC score	23 (8)	23 (9)
Pre-morbid Katz score		
Low score (0-3)	2 (2%)	3 (3%)
High score (4-6)	90 (98%)	109 (97%)
Body-mass index (kg/m^2)	29 (8)	27 (6)
ICU length of stay (days)*	7 (5)	7 (5)
Mode of ventilation*		
Invasive	110 (96%)	91 (97%)
Non-invasive	5 (4%)	3 (3%)
Duration of ventilation (days)*	5·7 (4·0)	5·4 (3·8)

***** At time of randomisation. Data are mean (SD) or n (%).

Table S3 Primary outcome measure after multiple imputation.

	Intensive n=150		Standard care n=158		Difference*
	Physical Component Summary measure of SF-36				
6 months	72/107	37 (11.4)	68/102	37 (10.2)	-0.7 (-5·1–3.7)

Imputations for PCS were made using predictive mean matching (PMM) method with 20 iterations.
* Adjusted difference in means (95% CI). Multiple linear regression models included stratification variables (unit, admission type and pre-admission Katz Index) and baseline variables sex, mode of ventilation, specialty, age in years, BMI, randomisation lag (time between admission to ICU and participant randomisation), duration of ventilation, and ICNARC Physiology Score.

Table S4 Health economic analysis

	Intensive		Standard care		Difference
Resource use during primary hospital admission					
ICU length of stay (days)	n=150	18 (18)	n=158	16 (14)	1·9 (-1·7–5·5)
Hospital ward length of stay (days)	n=124	42 (38)	n=118	41 (38)	0·6 (-9·0–10·2)
Total physiotherapist time (minutes)	n=134	348 (315)	n=137	210 (229)	138 (73–204)
Resource use during follow-up					
Primary care (visits)					
Practice	n=21	3·7 (4·5)	n=20	2·3 (2·2)	1·4 (-0·8–3·7)
Home	n=20	0·9 (1·4)	n=22	1·1 (1·5)	-0·2 (-1·2–0·7)
Telephone	n=21	1·0 (2·3)	n=21	1·0 (1·0)	0·5 (-1·0–1·1)
Practice nurse	n=15	6·4 (15·0)	n=10	11 (21·.5)	-4·6 (-19·7–10·5)
Hospital specialist (visits)	n=21	3·0 (3·3)	n=17	3·2 (3·7)	-0·2 (-2·5–2·1)
Hospital length of stay (days)	n=6	7·7 (10·3)	n=8	24·2 (39·9)	-16·6 (-53·3–20·1)
Prescriptions (times)	n=15	3·1 (2·2)	n=11	2·5 (1·8)	0·7 (-1·0–2·3)
Utility values					
SF-6D	n=53/107		n=49/102		
Baseline utility score		0·301		0·301	-
3 months utility score		0·603 (0·145)		0·561 (0·181)	0·042 (-0·022–0·106)
6 months utility score		0·641 (0·173)		0·579 (0·217)	0·061 (-0·016–0·138)
QALY over 6 months		0·269 (0·055)		0·250 (0·070)	0·018 (-0·006–0·043)
EQ-5D	n=55/107		n=56/102		
Baseline utility score		0·075		0·075	-
3 months utility score		0·512 (0·353)		0·446 (0·324)	0·065 (-0·062–0·193)
6 months utility score		0·565 (0·390)		0·502 (0·347)	0·064 (-0·075–0·202)
QALY over 6 months		0·208 (0·127)		0·184 (0·116)	0·024 (-0·022–0·070)

Data are mean (SD). Total physiotherapist time includes the time spent by all physiotherapists to deliver the physical rehabilitation sessions in the trial; some sessions may have required two or more physiotherapists. Baseline utility values were not collected but were assumed to be the same low value for all participants. Resource use during follow-up was based on a 32 questionnaires returned from participants in each group (total 64 questionnaires).

Abstract presented at Chartered Society of Physiotherapists Congress, Liverpool, 15-16 October 2010

Title: A survey to evaluate the provision of critical care physiotherapy services in the North East of England

Presenting author: Georgina Temple (Senior Physiotherapist)
Co-authors: Catherine Baker (Clinical Lead Physiotherapist)
Dr Zubair Umer Mohamed (Specialist Registrar)
Dr Stephen Wright (Consultant Intensivist)
Address: Newcastle Upon Tyne Hospitals NHS Trust, Newcastle upon Tyne, UK

Presentation Category: Special Interest Reports

Presentation Format: Poster Presentation

Purpose:
This survey was undertaken to evaluate the provision of physiotherapy services to critically ill patients in the North East of England.

Relevance:
Following the publication of NICE clinical guideline 83 - Rehabilitation after critical Illness, many Critical Care Units (ICUs) have reflected on their own physiotherapy and rehabilitation services. Gap analysis within our own Trust showed significant shortcomings compared to the standards recommended in the guideline. A region-wide evaluation will provide useful comparative data for physiotherapy teams working in critical care.

Description:
We designed a questionnaire to evaluate four key areas: manpower; frequency and type of physiotherapy interventions; current follow-up services; and changes since NICE Guideline 83. The lead physiotherapist for each of the 14 adult ICUs in our region was contacted by telephone and a questionnaire completed for all units. Data were analysed using Microsoft Excel 2003.

Evaluation:
The median number of critical care beds per whole time equivalent (WTE) physiotherapist was 9 (range 5-16); the NICE recommendation is 4 beds. The most common frequency of interventions (e.g. standing practice and general limb strengthening) was once-daily, Monday to Friday. In some ICUs, physiotherapists provide twice-daily interventions, 7 days per week. One hospital in our region has a post-ICU rehabilitation programme. Since NICE guideline 83, one third of ICUs have seen minor changes in their physiotherapy services with improved documentation, handover, and use of rehabilitation goals.

Conclusion:
The provision of physiotherapy services was very variable across our region. Post-ICU rehabilitation programmes are rare. As yet, we have seen few changes in critical care physiotherapy since the publication of national guidelines.

Implications:
These data will be useful for both local planning and to allow regional comparison of the provision of critical care physiotherapy services. Significant financial investment would be required in our region to achieve the standards recommended by NICE 83.

Keywords: NICE clinical guideline 83
Rehabilitation
Critical Illness

Funding: Unfunded

Ethics: This survey fulfils the National Research Ethics Service (NRES) criteria for service evaluation and so did not require ethical approval.
